# The Density and Length of Root Hairs Are Enhanced in Response to Cadmium and Arsenic by Modulating Gene Expressions Involved in Fate Determination and Morphogenesis of Root Hairs in *Arabidopsis*

**DOI:** 10.3389/fpls.2016.01763

**Published:** 2016-11-23

**Authors:** Ramin Bahmani, Dong G. Kim, Jin A. Kim, Seongbin Hwang

**Affiliations:** ^1^Department of Molecular Biology, Sejong UniversitySeoul, South Korea; ^2^Department of Bioindustry and Bioresource Engineering, Sejong UniversitySeoul, South Korea; ^3^Plant Engineering Research Institute, Sejong UniversitySeoul, South Korea

**Keywords:** root hair, cadmium, arsenic, gene expressions, *Arabidopsis*

## Abstract

Root hairs are tubular outgrowths that originate from epidermal cells. Exposure of *Arabidopsis* to cadmium (Cd) and arsenic [arsenite, As(III)] increases root hair density and length. To examine the underlying mechanism, we measured the expression of genes involved in fate determination and morphogenesis of root hairs. Cd and As(III) downregulated *TTG1* and *GL2* (negative regulators of fate determination) and upregulated *GEM* (positive regulator), suggesting that root hair fate determination is stimulated by Cd and As(III). Cd and As(III) increased the transcript levels of genes involved in root hair initiation (*RHD6* and *AXR2*) and root hair elongation (*AUX1*, *AXR1*, *ETR1*, and *EIN2*) except *CTR1. DR5*::*GUS* transgenic *Arabidopsis* showed a higher *DR5* expression in the root tip, suggesting that Cd and As(III) increased the auxin content in the root tip. Knockdown of *TTG1* in *Arabidopsis* resulted in increased root hair density and decreased root hair length compared with the control (Col-0) on 1/2 MS media. This phenotype may be attributed to the downregulation of *GL2* and *CTR1* and upregulation of *RHD6*. By contrast, *gem* mutant plants displayed a decrease in root hair density and length with reduced expression of *RHD6*, *AXR2*, *AUX1*, *AXR1*, *ETR1*, *CTR1*, and *EIN2*. Taken together, our results indicate that fate determination, initiation, and elongation of root hairs are stimulated in response to Cd and As(III) through the modulation of the expression of genes involved in these processes in *Arabidopsis*.

## Introduction

The metalloid arsenic (As) and the heavy metal cadmium (Cd) are highly toxic elements present in the environment, and they pose a considerable risk to living organisms owing to their accumulation in the soil and water (Godt et al., 2006; Zhao et al., 2009). Typical toxic effects of Cd on plants are the inhibition of primary root growth and increased lateral root density (Hu et al., 2013). Although the influence of Cd on root hairs has not been researched in detail, studies have shown that Cd at specific concentrations (that inhibit root growth by about 50%) increases root hair density and length in various plant species including radish (Vitoria et al., 2003), barley (Durcekova et al., 2007), sorghum (Kuriakose and Prasad, 2008), and Rhode grass (Kopittke et al., 2010). However, the molecular mechanism of root hair enhancement in response to Cd and As has not been reported to date.

Root hairs are tubular outgrowths from specific epidermal cells that function in nutrient and water absorption (Peterson and Farquhar, 1996). In *Arabidopsis* (*Arabidopsis thaliana*), root anatomy is well determined, and the different steps of root hair development including fate determination, initiation, bulge formation, and tip growth have been defined (Schiefelbein and Somerville, 1990; Dolan et al., 1993; Galway et al., 1994). By definition, epidermal cells located over the anticlinal junction between two underlying cortical cells differentiate into trichoblast cells that develop root hairs, while epidermal cells that lie above the outer periclinal cortical cells become non-hair cell atrichoblasts (Masucci et al., 1996; Dolan, 2006).

In *Arabidopsis*, several genes and transcription factors are involved in epidermal cell fate determination. Previous studies showed that root epidermal cells differentiate into non-hair cells through the action of the transcriptional complex WER-GL3/EGL3-TTG1, which consists of the R2-R3 MYB transcription factor WER (WEREWOLF), the basic Helix-Loop-Helix (bHLH) transcription factors GL3 (GLABRA3) and EGL3 (ENHANCER OF GLABRA3), and TTG1 encoding a protein of 341 amino acid residues containing four WD-40 repeats. By contrast, a root hair cell is produced if the transcriptional complex CPC/TRY/ETC1 (CAPRICE/TRIPTYCHON/ENHANCER OF TRY AND CPC1, one-repeat Myb protein)-GL3/EGL3-TTG1 is present in the epidermal cell (Wada et al., 1997; Lee and Schiefelbein, 2002; Schellmann et al., 2002; Kirik et al., 2004; Simon et al., 2007; Benitez and Alvarez-Buylla, 2010). The generation of each complex depends on the relative abundance of two competing transcription factors, WER/MYB23 and CPC/TRY/ETC1 (Wada et al., 1997; Lee and Schiefelbein, 1999). In non-hair cells, high expression levels of *WER* make the complex to be mainly an active form, which promotes the expression of *GL2* (GLABRA2, homeodomain transcription factor) and the repression of *RHD6* (ROOT HAIR DEFECTIVE 6, bHLH transcription factor) transcription, suppressing the expression of atrichoblast-specific genes and preventing root hair formation (Masucci and Schiefelbein, 1994, 1996; Rerie et al., 1994; Lee and Schiefelbein, 1999; Menand et al., 2007; Bruex et al., 2012). On the other hand, in root hair cells, the perception of a JACKDAW (JKD, zinc finger transcription factor)-induced positional signal in the cortex by SCM (SCRAMBLED, leucine-rich repeat receptor-like kinase) inhibits *WER* expression (Kwak et al., 2005; Hassan et al., 2010). Eventually, reduced *WER* expression as well as competitive inhibition of WER binding to GL3/EGL3-TTG1 by CPC/TRY/ETC1 (Lee and Schiefelbein, 2002) lead to the formation of an inactive complex, CPC-GL3/EGL3-TTG1, and the repression of *GL2* and root hair cell differentiation. The WER-GL3/EGL3-TTG1 complex positively regulates the transcription of CPC/TRY/ETC1 and negatively regulates that of GL3/EGL3 in atrichoblast cells. Therefore, it was proposed that CPC/TRY/ETC1 translocates from non-hair cells to hair cells and GL3/EGL3 in the opposite direction via plasmodesmata (Kurata et al., 2005; Kang et al., 2013).

Genetic analysis of root hair mutants demonstrated that the phytohormones ethylene and auxin regulate root hair formation independently or downstream of the WER-GL3/EGL3-TTG1 or CPC-GL3/EGL3-TTG1 pathway (Schiefelbein, 2000; Muller and Schmidt, 2004). For instance, *ROOT HAIR DEFECTIVE6* (*RHD6*), a downstream gene of *GL2*, encodes a bHLH transcription factor (Qing and Aoyama, 2012) and its mutation results in a decrease of root hair number as well as a shift in the position of root hair emergence (Masucci and Schiefelbein, 1994; Menand et al., 2007). However, this defect can be reversed by the application of auxin (IAA) or ACC (ethylene precursor), implying that *RHD6* plays a role in root hair formation (probably initiation) via auxin and/or ethylene related pathways. Mutations in the *AXR2* (AUXIN RESISTANT2) gene, which encodes the Aux/IAA protein, result in a decline in root hair number (Pitts et al., 1998; Nagpal et al., 2000; Rigas et al., 2013). *AXR2* represents a converging point of the auxin and ethylene pathways, since treatment with IAA or ACC does not recover root hair numbers in *axr2* mutants (Masucci and Schiefelbein, 1996). Mutants of the *AUX1* gene, which encodes an auxin influx carrier, display shorter root hairs (Pitts et al., 1998; Schmidt and Schikora, 2001; Yu et al., 2015). Mutants of *AXR1* (AUXIN RESISTANT1), which encodes a ubiquitin-activating enzyme E1, have shorter root hairs than the WT (Cernac et al., 1997; Pitts et al., 1998). Regarding ethylene, root hair formation is suppressed by the ethylene biosynthesis inhibitor AVG (Tanimoto et al., 1995; Masucci and Schiefelbein, 1996) and enhanced by ACC (ethylene precursor) treatment (Tanimoto et al., 1995). Nevertheless, the role of ethylene in root hair formation remains unclear, since the ethylene signaling mutants *etr1* (ETHYLENE RESPONSE1) and *ein2* (ETHYLENE INSENSITIVE2) show normal root hair density (Masucci and Schiefelbein, 1996). However, *ein2* mutants have shorter root hairs (Rahman et al., 2002). In addition, the ethylene response mutant *ctr1* (CONSTITUTIVE TRIPLE RESPONSE1), which is constitutively active in ethylene signal transduction, possesses a greater root hair density (Masucci and Schiefelbein, 1996), and the ethylene overproducing mutant *eto1* exhibits longer hairs (Strader et al., 2010) than the WT. Furthermore, ethylene promotes auxin biosynthesis and transport, implying that ethylene may be involved in root hair formation through the auxin pathway (Ruzicka et al., 2007; Swarup et al., 2007).

In the present work, we examined the enhancement of root hair density and length by Cd and As(III) based on the hypothesis that root hair fate determination and morphogenesis are affected by cadmium and arsenic. Our results suggested that cadmium and arsenic increase root hair density and length by modulating the expression of genes involved in fate determination and the initiation and elongation of root hairs. Based on our results, we proposed a model in which cadmium and arsenic enhance root hair density and length by promoting the convergence of developmental (*TTG1*/*GL2/GEM*) and hormonal (auxin and ethylene) signaling pathways.

## Materials and Methods

### Plant Material and Heavy Metal Treatment

Seeds of *Arabidopsis* (*A. thaliana* L. Heynh) ‘Columbia’ ecotype, *ttg1* (SALK_054584), and *gem* (SALK_145846C) were obtained from the *Arabidopsis* Biological Resource Center (Ohio State University) and used in the study. Seeds were surface-sterilized by immersion in 95% ethanol for 3 min and a 20% bleach solution containing 0.1% Tween 20 for 5 min, followed by at least three rinses in ddH_2_O (sterile Milli-Q water). Then, seeds were germinated and grown on agar plates containing 1/2 MS nutrient medium (Duchefa) and 1% sucrose, with pH adjusted to 5.7 by KOH, and 0.5% agar in a growth chamber at 23°C with 60% relative humidity in a long day photoperiod (16 h light/8 h dark) in a vertical position. In each experiment, 10 seeds were cultured on a 90-mm Petri dish containing 20 mL of medium. Aliquots of 100 mM CdSO_4_ and AsNaO_2_ stock solution for cadmium and arsenic media were added to the 1/2 MS media after autoclaving to result in final concentrations of 40 μM Cd and 10 μM As(III).

### Morphometric Analysis

After 1 week of growth, *Arabidopsis* seedlings were viewed under a Leica stereoscopic microscope (MZ10F, Germany). Photographs were taken using a DFC450 C (Leica, Germany) camera under appropriate magnification (5–40×). Root hair density was determined as the number of hairs in a 5 mm section from the starting point of the root differentiation zone under a microscope (40×). Root hair length was measured in a 5 mm section from the starting point of the root differentiation zone and quantified using Image J software (National Institutes of Health^1^). Results were expressed as the mean ± standard error (SE). All measurements indicate the average of three independent experiments, each involving 10 seedlings.

### Quantitative RT-PCR Analysis

For gene expression analysis, total RNA was isolated from *Arabidopsis* plants grown on 1/2 MS agar plates supplemented with or without Cd and As(III) for 1 week using the TRIzol reagent (Invitrogen, USA) according to the manufacturer’s instructions. The first strand cDNA was synthesized from 2 μg of each RNA sample using the cDNA Synthesis Kit (Wizbio, Korea). qRT-PCR was performed using a CFX Connect^TM^ Real-Time PCR Detection System (Bio-Rad Laboratories Inc., Hercules, CA, USA). Each 20 μL reaction mixture consisted of 10 μL of SYBR Supermix (SsoAdvanced^TM^ Universal SYBR^®^ Green Supermix), 1 μL of cDNA, 7 μL of nuclease-free water, and 1 μL of each primer. The qRT-PCR reaction thermal conditions were as follows: 95°C for 30 s, 40 cycles at 95°C for 15 s, and 60°C for 20 s. All samples were run in triplicate (three biological repeats) for cDNA and each primer set. Relative transcript levels were assessed by normalization to *actin* transcript abundance as an endogenous control. The gene specific primers are listed in **Supplementary Table S1**.

### Semi-Quantitative RT-PCR

Total RNA was isolated from *Arabidopsis* plants grown on 1/2 MS agar plates with and without Cd and As(III) for 1 week using the TRIzol reagent (Invitrogen), and RT-PCR was performed using 250 ng of total RNA with an RT-PCR kit (Takara) as follows: 30 cycles of 30 s at 95°C, 30 s at different annealing temperatures, 120 s at 72°C. Annealing temperatures were as follows: 60°C for *AtActin*; 58°C for *AtTTG1*, *AtGEM*, and *AtAXR2*; 51°C for *AtRHD6*, *AtAUX1*, and *AtAXR1*; 52°C for *AtGL2*; and 50°C for *AtEIN2*, *AtCTR1*, and *AtETR1*. The RT-PCR analysis was repeated at least five times using separately harvested samples. For semi-quantitative RT-PCR analysis, RT-PCR products were analyzed using ImageJ software to quantify the expression level of each gene. The gene-specific primers used for semi-quantitative RT-PCR are listed in **Supplementaty Table S2**.

### β-Glucuronidase Assay

GUS histochemical staining of *Arabidopsis* plants harboring *AtGL2*::*GUS* and *AtDR5*::*GUS* fusion constructs was performed as previously described (Prasinos et al., 2005) with minor modifications. Briefly, 5-day-old seedlings grown vertically on 1/2 MS agar medium supplemented with or without Cd (40 μM) and As(III) (10 μM) were immersed in GUS staining solution (50 mM NaPO_4_ buffer, pH 6.8, 0.5 M EDTA pH 8.0, 0.5 mM potassium ferrocyanide, 0.5 mM potassium ferricyanide, 20% triton X-100, and 2 mM X-gluc), and incubated overnight at 37°C. Photographs were taken with a microscope (Leica MZ10F, Germany) equipped with a digital camera (DFC450 C). GUS stained tissues represent the results of at least five independent seedlings.

### Statistical Analysis

One-way ANOVA was used to analyze the data using SAS software (version 9.1), and means were compared using Tukey’s multiple comparison test, with significant differences at *P* ≤ 0.05 or *P* ≤ 0.01.

## Results

### Root Hair Density and Length Were Increased in Response to Cd and As(III) in *Arabidopsis*

**FIGURE 1:**
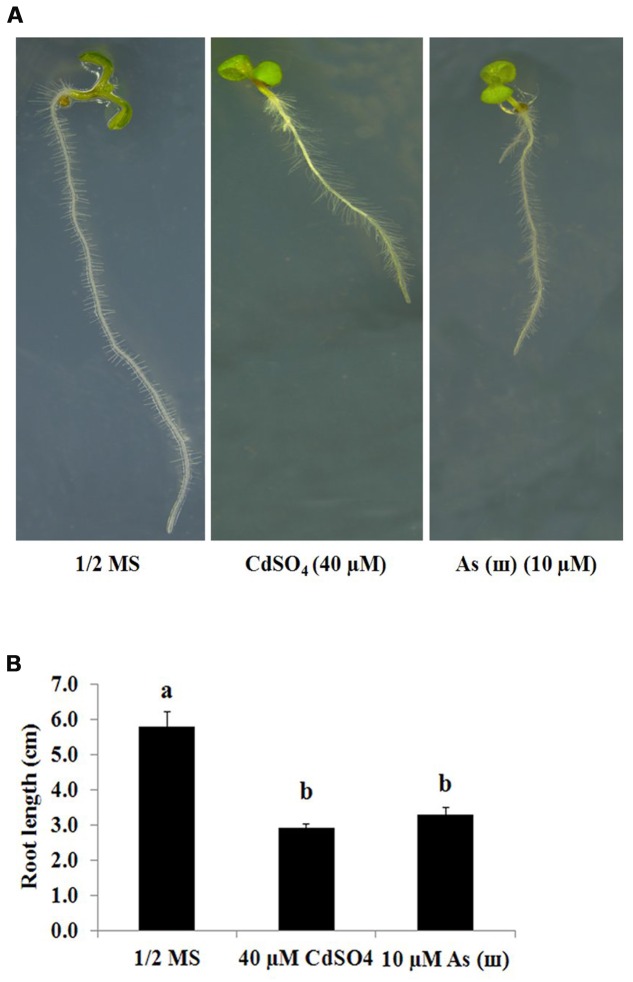
**Inhibitory effect of Cd (CdSO_4_) and As(III) (AsNaO_2_) stress on primary root growth in *Arabidopsis*.** **(A)** Morphology of 1-week-old seedlings germinated and grown on 1/2 MS agar plates containing no metal (left), 40 μM CdSO_4_ (center), and 10 μM AsNaO_2_ (right). **(B)** Quantification of the primary root length of *Arabidopsis* shown in **(A)**. Values are average (±SE) of three independent experiments, each involving 10 seedlings. Different letters in each column indicate a significant difference according to Tukey’s multiple comparison test (*p* ≤ 0.01).

**FIGURE 2:**
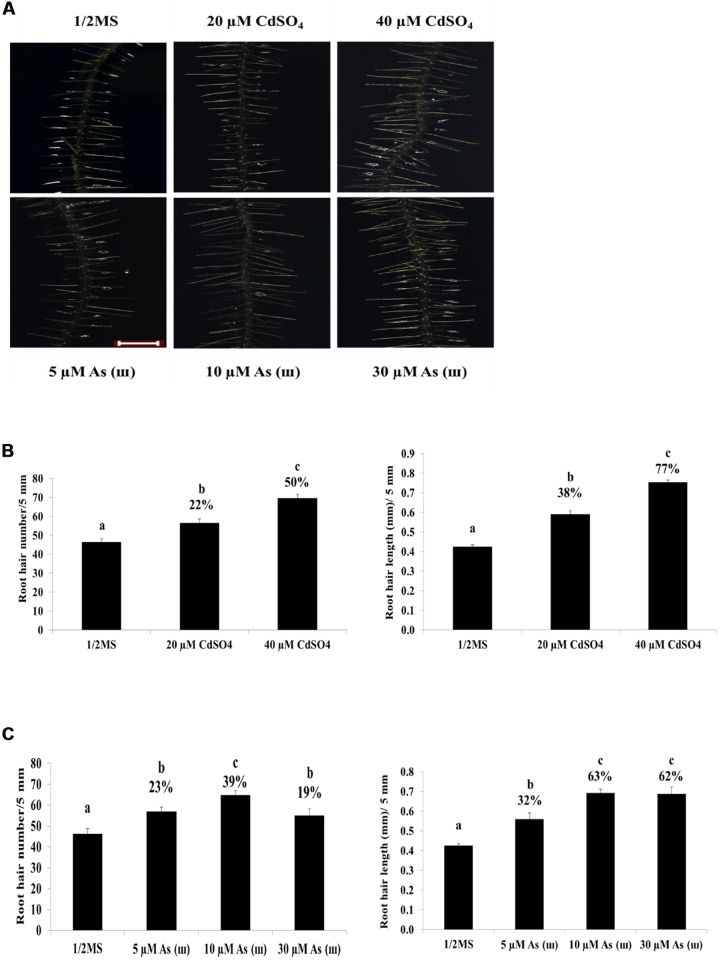
**Effect of Cd and As(III) on the length and density of root hairs in *Arabidopsis*.** **(A)** Root hair morphology of *Arabidopsis Col*-0 seedlings germinated and grown for a week on 1/2 MS agar media treated with no metal, 20, 40 μM CdSO_4_, and 5, 10, 30 μM As(III). Roots were observed at 40× magnification; scale bars = 0.2 mm. Photographs show plants representative of the 10 plants examined in each experiment. **(B)** Density and length of root hair treated with Cd shown in **(A)**. Root hair density was expressed as the number counted in a 5 mm section from the starting point of differentiation zone under a microscope. Root hair length was measured in a 5 mm section of differentiation zone. Root hairs were photographed with Leica Stereoscopic microscope (MZ10F, Germany), and their length was quantified using Image J software as described in Materials and methods. Values are average (±SE) of three independent experiments, each involving 10 seedlings. **(C)** Density and length of root hairs treated with As(III) shown in **(A)**. Root hair density and length were measured as in **(B)**. Different letters in each column indicate a significant difference according to Tukey’s multiple comparison test (*p* ≤ 0.01).

*Arabidopsis* plants grown on 1/2 MS agar medium containing Cd (CdSO_4_, 40 μM) and AsIII (AsNaO_2_, 10 μM) for 1 week showed a 50% decrease in primary root length (**Figures 1A,B**). By contrast, root hair length and density increased significantly in *Arabidopsis* plants exposed to Cd and As(III) compared with those in control plants (**Figure 2A**). Exposure to Cd at 20 and 40 μM increased the number of root hairs in a 5 mm section from the starting point of the root differentiation zone by 22 and 50%, respectively, compared with control plants (**Figure 2B**). Root hair number and length could not be measured at 60 μM Cd because germination was completely inhibited (data not shown). As(III) treatment increased the number of root hairs by 23% at 5 μM, 39% at 10 μM, and 19% at 30 μM (**Figure 2C**). Cd-treated plants showed an increase in root hair length of 38% at 20 μM and 77% at 40 μM compared with that of the control (1/2 MS) (**Figure 2B**). As(III) treatment also increased root hair length by 32% at 5 μM, 63% at 10 μM, and 62% at 30 μM (**Figure 2C**).

To determine whether the metal-induced increase in root hair density was related to the reduction of root cell length, we measured root epidermal cell length in control and metal-treated plants. The root cell length of control plants was 151.7 μm, whereas that of Cd and As(III)-treated plants was 140.38 and 144.2 μm, respectively (**Supplementary Figure S4**). These results indicated that the increase in root hair number was not associated with changes in root cell length.

### Cd and As(III) Modulated the Expression of Genes Involved in Root Hair Fate Determination (Differentiation)

**FIGURE 3:**
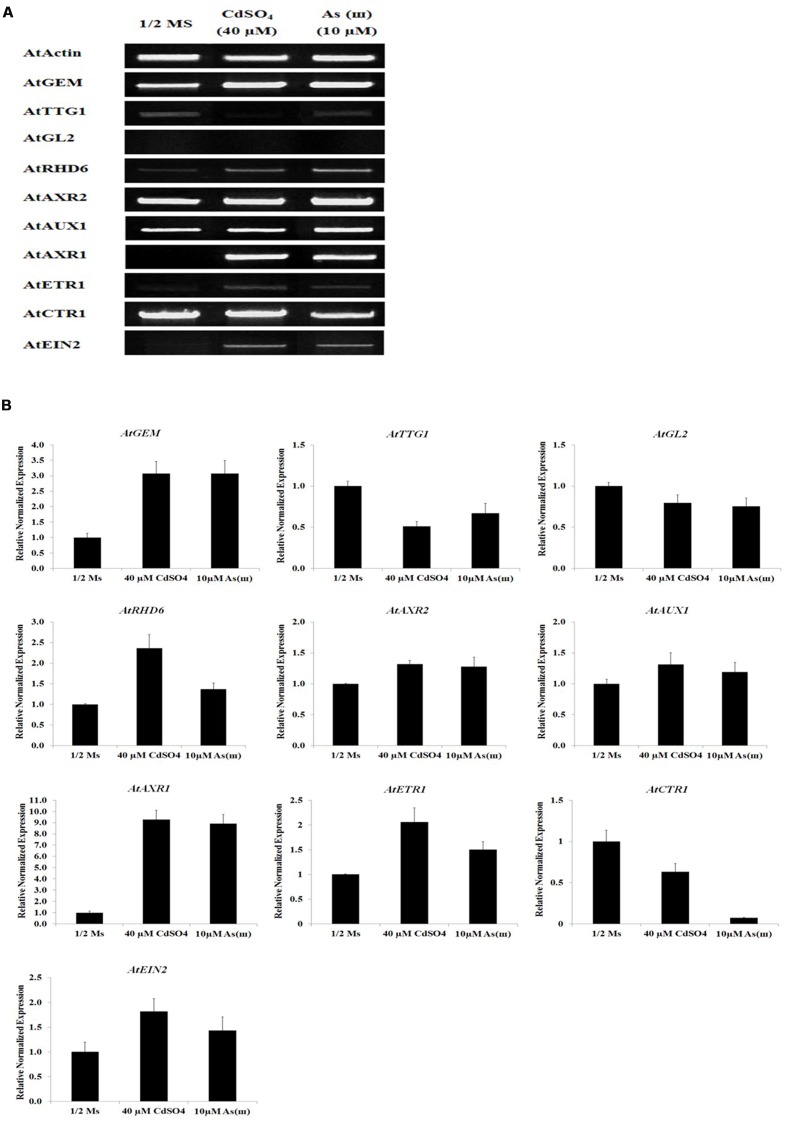
**Effect of Cd and As(III) on expressions of genes involved in the differentiation (fate determination) and elongation of root hairs in *Arabidopsis*.** **(A)** Semi-quantitative RT-PCR results showing relative transcript levels of various genes involved in the differentiation (*GEM*, *TTG1*, and *GL2*), initiation (*RHD6* and *ACXR2*) and elongation (*AUX1*, *AXR1*, *ETR1, CTR1*, and *EIN2*) of root hairs in response to Cd and As(III) in *Arabidopsis*. RT-PCR was performed using total RNA isolated from 1-week-old plants which were germinated and grown on 1/2 MS agar media treated with no metal, 40 μM CdSO_4_ and 10 μM As(III).The gel photography represents 3 experimental replicates. **(B)** Quantitative real-time PCR results of various genes involved in root hair differentiation and elongation shown in **(A)**. Transcript levels were normalized to the transcript level of *Actin*.

**FIGURE 4:**
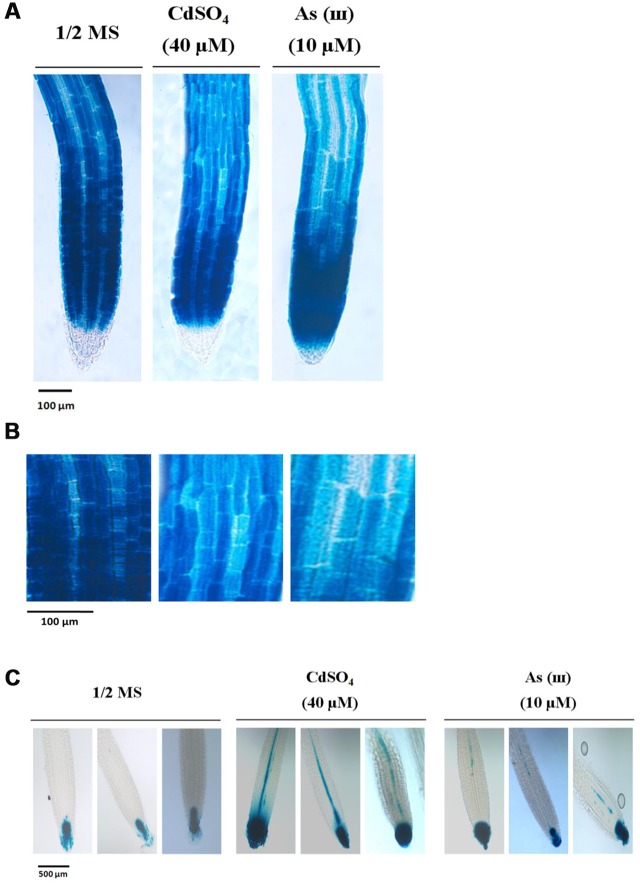
**Down-regulation of *GL2* and up-regulation of *DR5* by cadmium and arsenite in *Arabidopsis* root.** **(A)** GUS activity of primary root tips (2 mm) of 5-day-old transgenic *Arabidopsis Col*-0 seedlings carrying the *GL2-GUS* construct. Plants were germinated and grown in 1/2 MS media supplemented with or without cadmium and arsenite, and were stained to visualize GUS activity (20× magnification). **(B)** GUS activity at higher magnification (40×). Left panel, 1/2 MS media; central panel, 1/2 MS media with 40 μM CdSO_4_; right panel, 1/2 MS media with 10 μM As(III). Scale bar indicates a 200 μm in size. Each photograph represents five plants. **(C)** GUS activity of primary root tips (2.3 mm) of 5-day-old transgenic *Arabidopsis Col*-0 seedlings carrying the *DR5-GUS* construct. Plants were germinated and grown in 1/2 MS media supplemented with or without cadmium and arsenite, and were stained to visualize GUS activity (20× magnification). Left panel, 1/2MS media; central panel, 1/2 MS media with 40 μM CdSO_4_; right panel, 1/2 MS media with 10 μM As(III). Scale bar indicates a 500 μm in size. Each photograph represents five plants.

Our findings that exposure to metals increased root hair density and length suggested that Cd and As(III) promoted the differentiation and elongation of root hairs. Developmental pathways regulated by the *TTG1*/*GL2* interaction determine root epidermal cell fate (Masucci and Schiefelbein, 1996). We therefore measured the expression levels of *TTG1* and *GL2* using semi-quantitative RT-PCR (**Figure 3A**, graphs in **Supplementary Figure S1**), qRT-PCR (**Figure 3B**), and GUS staining (**Figures 4A,B**). Cd and As(III) markedly downregulated *TTG1* expression compared with that in the control, providing an explanation for the increment in root hair density induced by Cd and As(III), since *TTG1* is a negative regulator of root hair differentiation (**Figures 3A,B**). The expression of *GL2*, another negative regulator of root hair differentiation, was also repressed by Cd and As(III). In addition, GUS activity in *Arabidopsis* plants expressing the *GL2*::*GUS* construct which were germinated and grown for 5 days on medium containing Cd and As(III) decreased compared with that in controls, suggesting that *GL2* expression is downregulated by Cd and As(III) (**Figures 4A,B**). This finding is contrary to previous studies showing that *GL2-GUS* is preferentially expressed in developing atrichoblast cells within the meristematic and elongation zones of the *Arabidopsis* root (Masucci et al., 1996). Taken together, these results indicated that the Cd or As(III) induced increase in root hair density was probably caused by the inhibition of *GL2* and *TTG1* gene expression. Because GEM plays a positive role in root hair fate determination (Caro et al., 2007), we examined the expression of *GEM* in Cd and As(III) treated plants. We found that the two metals significantly increased *GEM* mRNA levels compared with those in control untreated plants (**Figures 3A,B**). Taken together, our results suggested that Cd and As(III) increased root hair density by promoting root hair differentiation through the repression of *TTG1* and *GL2* (negative regulators of root hair differentiation) and the upregulation of *GEM* expression (positive regulator).

### Cd and As(III) Modulated the Expression of Genes Involved in Root Hair Initiation and Elongation (Auxin/Ethylene Signaling Pathway)

Several plant hormones are involved in root hair development (Lee and Cho, 2013). Among these, auxin and ethylene have been studied extensively (Masucci and Schiefelbein, 1996; Pitts et al., 1998; Cho and Cosgrove, 2002). Given the important role of auxin and ethylene in root hair initiation and elongation, we examined whether the expression of genes related to these hormones was affected by Cd and As(III).

The *RHD6* gene, a downstream gene of *GL2*, plays a key role in root hair initiation, as shown by the decreased root hair density and shift in the root hair initiation site observed in *rhd6* mutants (Masucci and Schiefelbein, 1994; Menand et al., 2007). Assessment of *RHD6* transcript levels in *Col*-0 *Arabidopsis* showed that Cd and As(III) upregulated *RHD6* expression (**Figures 3A,B**), suggesting that root hair initiation is promoted by Cd and As(III) through the enhancement of *RHD6* expression.

Next, we analyzed the expression of auxin-related genes in plants grown in media containing Cd and As(III). As shown in **Figures 3A,B**, *AXR2*, which functions in root hair initiation, and *AUX1* and *AXR1*, which are involved in root hair elongation, were upregulated in response to Cd and As(III). In addition, *DR5*::*GUS* transgenic *Arabidopsis* showed that auxin-inducible *DR5* expression was enhanced by Cd and As(III), indicating that the auxin level at root tip is increased by Cd and As(III) (**Figure 4C**). The expression of *DR5-GUS* around the vascular cylinder indicates that auxin is translocating to the root tip. Taken together, these results suggested that the auxin signaling pathway is activated in response to Cd and As(III), leading to root hair elongation.

The result that Cd and As(III) upregulated the expression of the auxin-resistant gene *AXR1* indicated that crosstalk between auxin and ethylene may be involved in metal-induced root hair elongation, since *AXR1* is upregulated by ethylene (Swarup et al., 2007). This raised the question of whether the change in transcript levels also occurs in ethylene signaling genes. To address this question, the mRNA levels of *ETR1*, *CTR1*, and *EIN2* were analyzed in Cd- and As(III)-treated plants. *ETR1* and *EIN2* were upregulated, whereas *CTR1*, a negative regulator of ethylene signaling, was downregulated by Cd and As(III). This indicated that the increase in root hair elongation in response to Cd and As(III) was mediated by the modulation of the expression of ethylene signaling genes (**Figures 3A,B**).

### Root Hair Density and *RHD6* Expression Were Enhanced, and Root Hair Length was Reduced in *ttg1* Mutants

**FIGURE 5:**
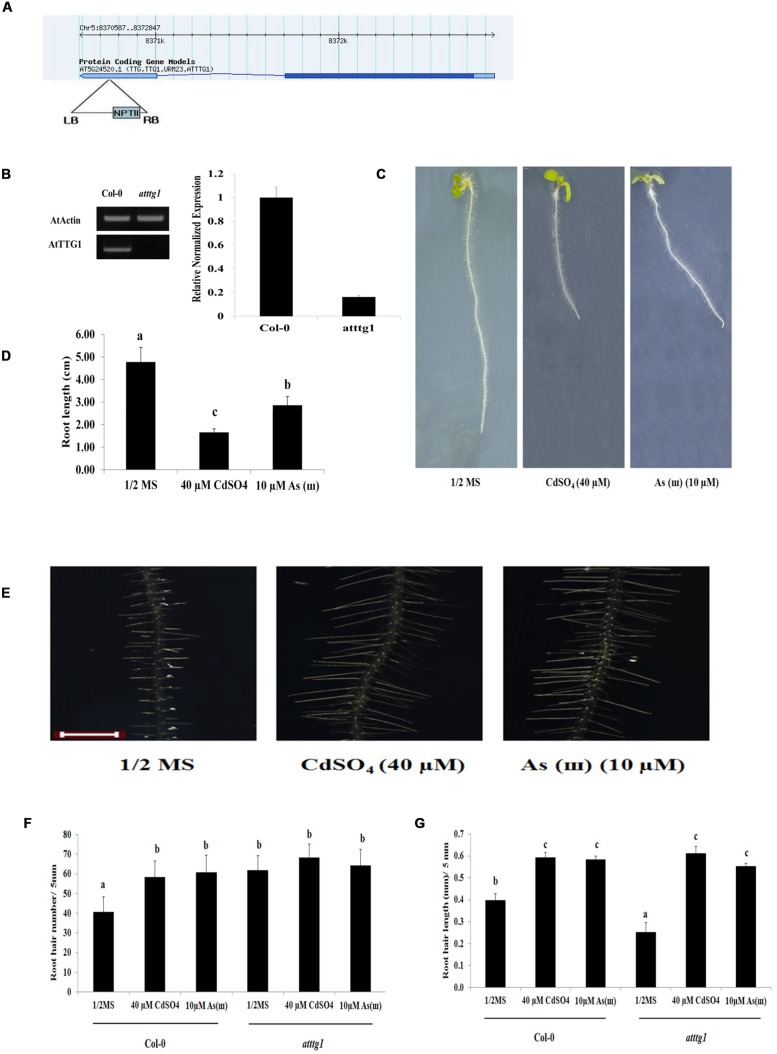
**Root hair length and density in response to Cd and As(III) in *ttg1 Arabidopsis*.** **(A)** Diagram showing T-DNA insertion position in *ttg1 Arabidopsis*. **(B)** Left, semi-quantitative RT-PCR products (transcript levels) of *AtTTG1* in control (*Col*-0) and *ttg1 Arabidopsis*. PCR product of actin (*AtActin*) was used as a loading control; Right, real-time PCR results. **(C)** Morphology of *ttg1 Arabidopsis* (1-week-old) seedlings germinated and grown on 1/2 MS agar plates containing no metal (left), 40 μM CdSO_4_ (center), and 10 μM AsNaO_2_ (right). **(D)** Quantification of the primary root length of *ttg1 Arabidopsis* shown in **(C)**. **(E)** Root hair morphology of *ttg1 Arabidopsis* germinated and grown on 1/2 MS with no metal (left), 40 μM CdSO_4_ (center) and 10 μM As(ø) (right). Roots were observed at 40× magnification; scale bars = 0.2 mm. **(F)** Graphs representing root hair density in a 5 mm section from the starting point of differentiation zone in *ttg1 Arabidopsis* shown in **(E)**. **(G)** Graphs representing root hair length in a 5 mm section from the starting point of differentiation zone in *ttg1 Arabidopsis* shown in **(E)**. Values are average (±SE) of three independent experiments, each involving 10 seedlings. Different letters over each column indicate a significant difference according to Tukey’s multiple comparison test (*p* ≤ 0.01).

The effects of Cd and As(III) on root hair development were also examined in the *ttg1* (negative regulator of root hair differentiation) mutant (**Figure 5**). *ttg1 Arabidopsis* (SALK_ 054584, **Figure 5A**) showed extremely low expression of *AtTTG1* (**Figure 5B**) and a decrease in root length in response to Cd and As(III) (**Figures 5C,D**). As shown in **Figures 5E,F**, root hair density was higher in *ttg1 Arabidopsis* plants than in control plants in 1/2 MS media, which is consistent with the role of *TTG1* as a negative regulator of root hair fate determination. However, root hair density was not enhanced by Cd and As(III), suggesting that the root hairs of the *ttg1* mutant were already fully differentiated to the level of metal-treated *Col*-0. By contrast, root hair length was slightly shorter in the *ttg1* mutant than in *Col*-0 in 1/2 MS medium, and it was increased by Cd and As(III) to the level of metal-treated *Col*-0. This implied that root hair differentiation was promoted, whereas root hair elongation was not enhanced in *ttg1*, confirming that *TTG1* is a negative regulator of root hair differentiation but not of root hair elongation.

**FIGURE 6:**
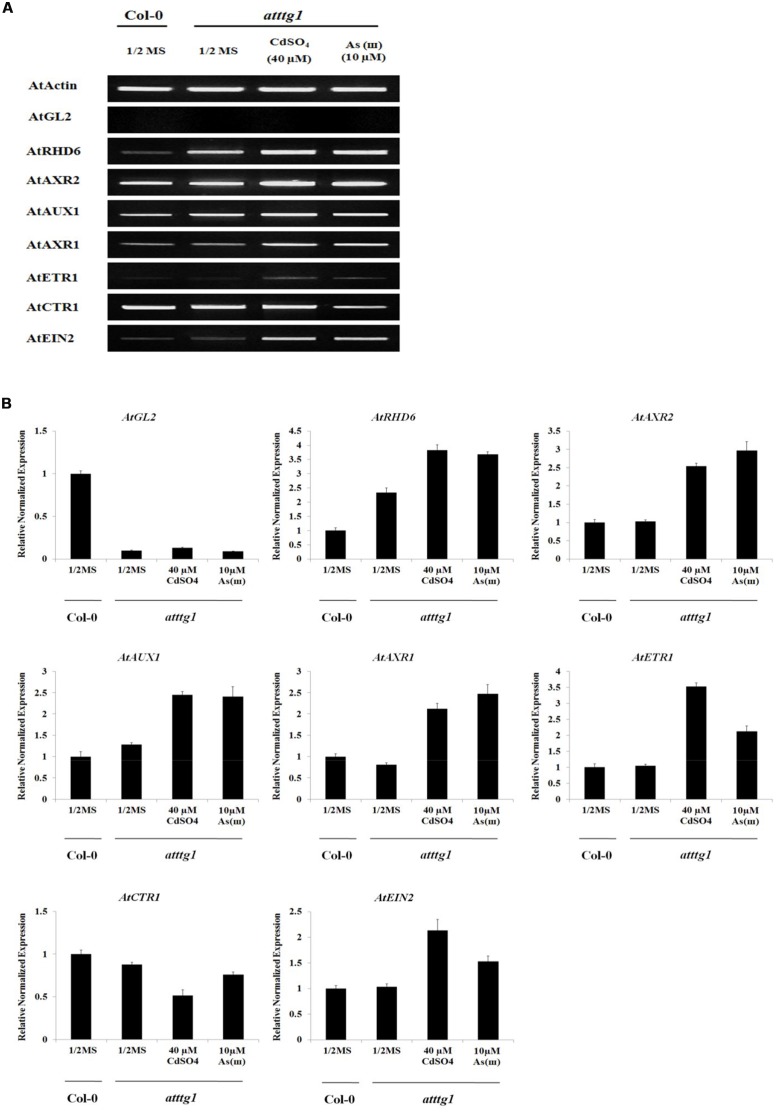
**Effect of Cd and As(III) on expressions of genes participated in the differentiation (fate determination) and elongation of root hairs in *ttg1 Arabidopsis*.** **(A)** Semi-quantitative RT-PCR results showing relative transcript levels of various genes involved in the differentiation (*GL2*), initiation (*RHD6* and *AXR2*) and elongation (*AUX1*, *AXR1*, *ETR1, CTR1*, and *EIN2*) of root hairs in *Col-0* and *ttg1 Arabidopsis*. RT-PCR was performed using total RNA isolated from 1-week old plants which were germinated and grown on 1/2 MS agar media treated with no metal, 40 μM CdSO_4_ and 10 μM As(III). The gel photography represents three experimental replicates. **(B)** Quantitative real-time PCR results of various genes involved in root hair differentiation and elongation shown in **(A)**. Transcript levels were normalized to the transcript level of *Actin*.

To understand the molecular mechanism underlying the enhanced root hair density and slightly reduced root hair length in *ttg1* plants, the expression of genes involved in fate determination, initiation, and elongation of root hairs was examined (**Figure 6** and **Supplementary Figure S2**). The expression of *GL2*, which was reported as a negative regulator of root hair fate determination, was repressed and not induced by Cd and As(III). This result supports previous reports that *GL2* is induced by *TTG1*, resulting in the suppression of root hair differentiation (Hung et al., 1998). In addition, the expression of *RHD6*, which is inhibited by *GL2* and involved in root hair initiation (Masucci and Schiefelbein, 1996), was upregulated in *ttg1* plants and this effect was enhanced by Cd and As(III). However, *AXR2* expression was not increased in *ttg1* plants, despite the fact that the *axr2* mutant has a lower number of root hairs (Masucci and Schiefelbein, 1996), implying that *AXR2* is involved in root hair initiation similar to *RHD6*. The expression of auxin/ethylene signaling genes (*AUX1*, *AXR1*, *ETR1*, *CTR1*, and *EIN2*) involved in root hair elongation in *ttg1* plants was similar to that in *Col*-0 plants, confirming that auxin/ethylene signaling genes are not controlled by *TTG1*/*GL2*. However, it was not clear why root hair length was shorter in *ttg1* than in *Col*-0, since the expression of root hair elongation genes was not decreased. Regarding root hair initiation and elongation in response to Cd and As(III), similar to *Col*-0, the expression of genes involved in root hair initiation (*RHD6* and *AXR2*) and elongation (*AUX1*, *AXR1*, *ETR1*, and *EIN2*) was increased, while *CTR1* expression was reduced.

### Root Hair Density and Length and the Expression of Genes Involved in Root Hair Initiation and Elongation Were Decreased in *gem* Mutants

**FIGURE 7:**
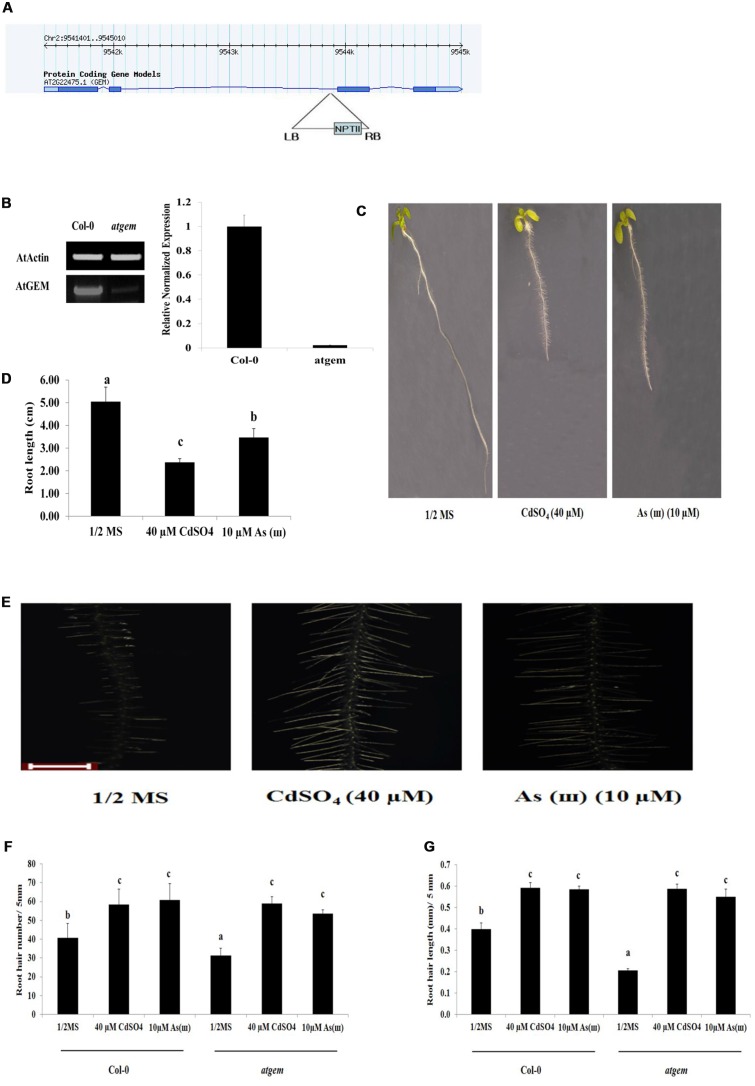
**Root hair length and density in response to Cd and As(III) in *gem Arabidopsis*.** **(A)** Diagram showing T-DNA insertion position in *gem Arabidopsis*. **(B)** Right, transcript levels (semi-quantitative RT-PCR) of *AtGEM* in control (*Col*-0) and *gem Arabidopsis*. A PCR product of actin (*AtActin*) was used as a loading control; Right, real-time PCR result. **(C)** Morphology of *gem Arabidopsis* (1-week-old) seedlings germinated and grown on 1/2 MS agar plates containing no metal (left), 40 μM CdSO_4_ (center), and 10 μM AsNaO_2_ (right). **(D)** Quantification of root length of *gem Arabidopsis* shown in **(C)**. **(E)** Root hair morphology of *gem Arabidopsis* germinated and grown on 1/2 MS with no metal (left), 40 μM CdSO_4_ (center), and 10 μM As(III) (right). Roots were observed at 40× magnification; scale bars = 0.2 mm. **(F)** Graphs representing root hair density in a 5 mm section from the starting point of differentiation zone in *gem Arabidopsis* root shown in **(E)**. **(G)** Graphs representing root hair length in a 5 mm section from the starting point of differentiation zone in *gem Arabidopsis* root shown in **(E)**. Values are average (±SE) of three-independent experiments, each involving 10 seedlings. Different letters in each column indicate a significant difference according to Tukey’s multiple comparison test (*p* ≤ 0.01).

Root hair density and length were also examined in *gem* (positive regulator of root hair fate determination) *Arabidopsis* mutants. As shown in **Figure 7**, both root hair density and length were decreased in *gem* plants (SALK_145846C) compared with those in control *Col*-0, whereas both were enhanced to the level of the control in response to Cd and As(III). This suggested that Cd and As(III) promoted root hair development in *gem* mutants by stimulating root hair differentiation (fate determination), initiation, and elongation.

**FIGURE 8:**
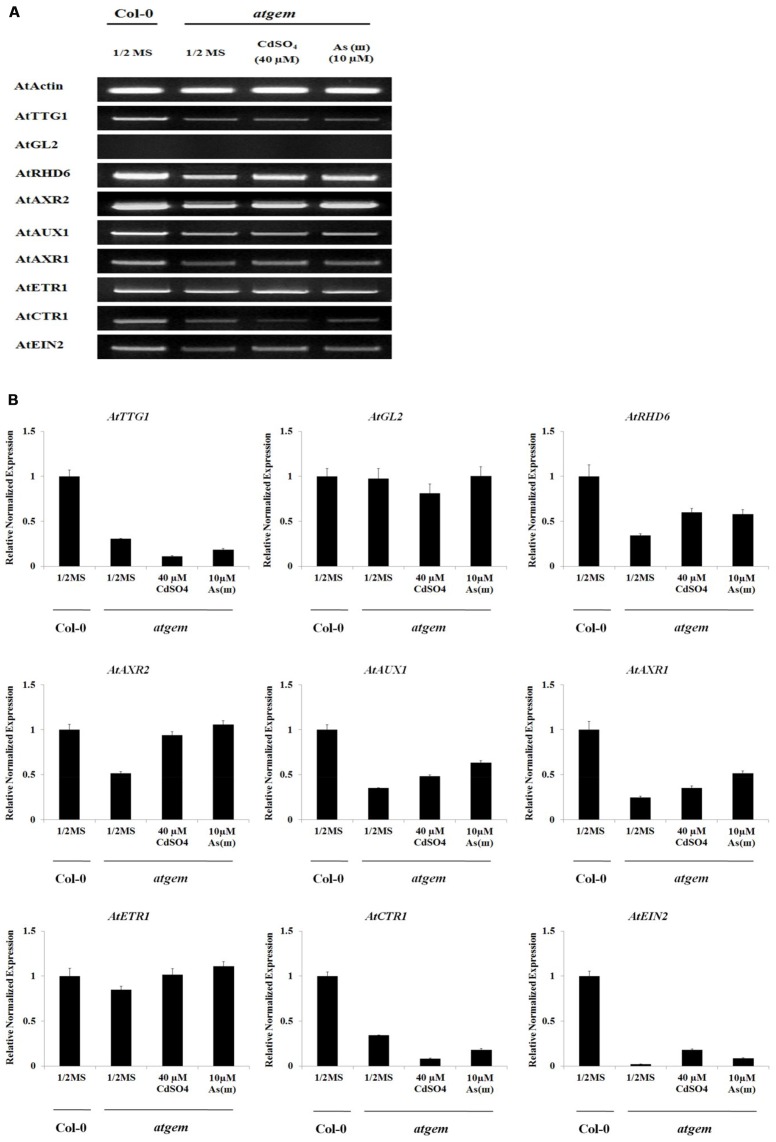
**Effect of Cd and As(III) on expressions of genes engaged in the differentiation (fate determination) and elongation of root hairs in *gem Arabidopsis*.** **(A)** Semi-quantitative RT-PCR results showing relative transcript levels of various genes involved in the differentiation (*TTG1* and *GL2*), initiation (*RHD6* and *ACXR2*) and elongation (*AUX1*, *AXR1*, *ETR1, CTR1*, and *EIN2*) of root hairs in *Col-0* and *gem Arabidopsis*. RT-PCR was performed using total RNA isolated from 1-week old plants which were germinated and grown on 1/2 MS agar media treated with no metal, 40 μM CdSO_4_ and 10 μM As(III). The gel photography represents three experimental replicates. **(B)** Quantitative real-time PCR results of various genes involved in root hair differentiation and elongation shown in **(A)**. Transcript levels were normalized to the transcript level of *Actin*.

To explain the Cd/As(III)-induced root hair development in *gem* plants, we examined the expression of genes involved in differentiation, initiation, and elongation of root hairs (**Figure 8** and **Supplementary Figure S3**). The expression of *TTG1*, a negative regulator of root hair differentiation, was lower in *gem* plants than in *Col*-0 and further downregulated by Cd and As(III). However, the expression of another negative regulator, *GL2*, was not altered. These results suggested that *GEM* is not a negative regulator of *TTG1* and *GL2*, which is in disagreement with previous reports indicating that *GEM* is a negative regulator of *TTG1*/*GL2* and promotes root hair differentiation (Caro et al., 2007). Furthermore, the lower expression of *TTG1* did not correlate with the reduced root hair density in *gem* plants, since *ttg1* plants displayed higher root hair density (**Figure 5F**). In addition, the downregulation of *RHD6* and *AXR2*, which function in root hair initiation, also contributed to the decrease of root hair density in *gem* plants.

Consistent with the decreased root hair length, the expression of genes involved in root hair elongation (*AUX1*, *AXR1*, *ETR1*, and *EIN2*) was inhibited in *gem* plants. Although Cd and As(III) upregulated these repressed genes, their expression did not recover to the level of *Col*-0 except *AXR2* and *ETR1*. It is beyond our expectation that *CTR1* expression was also repressed in *gem* plants, since *CTR1* is a blocker of ethylene signal transduction involved in root hair elongation.

## Discussion

**FIGURE 9:**
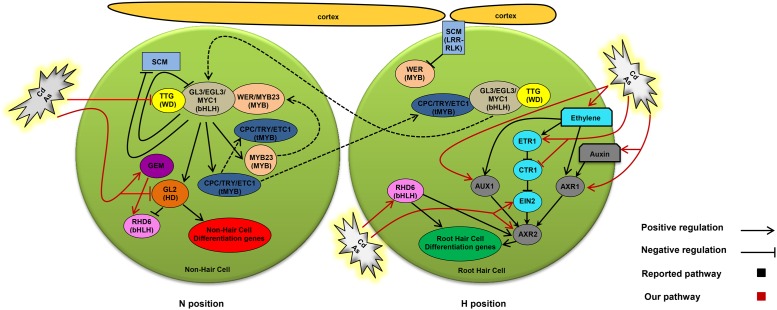
**Proposed model showing how cadmium and arsenic (As III) promote root hair differentiation and elongation through modulating intrinsic developmental and hormonal pathways in *Arabidopsis*.** Arrows show positive regulation, blunted lines show negative regulation, and broken lines illustrate intercellular or intracellular protein translocation. This model is adapted from Bruex et al. (2012).

In the present study, we showed that Cd and As(III) increased root hair density probably by converting non-hairy cells to hairy cells through the modulation of the expression of genes involved in root hair fate determination (**Figures 3** and **9**). First, Cd and As(III) downregulated *TTG1*, which is essential for generating the WER-GL3/EGL3-TTG1 complex; this impaired its ability to upregulate *GL2*, which resulted in cells entering the root hair cell fate pathway. Second, Cd and As(III) may inhibit *GL2* expression directly and render it unable to inhibit *RHD6* transcription, which is involved in hair initiation, as well as impairing the expression of atrichoblast-specific genes. Third, Cd and As(III) promoted *GEM* expression, positively regulating *RHD6* expression, which was downregulated in *gem Arabidopsis* (**Figure 8**); this led to the upregulation of root hair cell differentiation genes and the determination of hair cell fate.

The next step of root hair development may be root hair morphogenesis, including initiation and elongation. *RHD6* and *AXR2* are downstream components of fate determination that initiate root hair morphogenesis, since *rhd6* and *axr2* mutants have reduced root hair density (Masucci and Schiefelbein, 1994; Rigas et al., 2013). Our data showed that *RHD6* expression was increased by Cd and As(III), resulting in increased root hair density and length. *RHD6* expression was higher in the *ttg1* mutant, in which root hair density is increased, and was lower in the *gem* mutant, in which root hair number is reduced, than in the control. *AXR2* expression was enhanced by Cd and As(III), remained unchanged in *ttg1*, and was reduced in *gem* plants. Auxin and ethylene promote root hair initiation via *AXR2* independently of *RHD6*, since they rescue low root hair numbers in the *rhd6* mutant but not in the *axr2* mutant (Masucci and Schiefelbein, 1996). Although the *ctr1* mutant has a higher number of root hairs (Masucci and Schiefelbein, 1996), it is not clear whether it functions in hair initiation, since supporting data was not reported thereafter.

The auxin signaling components *AUX1* and *AXR1* are involved in root hair elongation since *aux1* and *axr1* mutants have shorter hairs (Pitts et al., 1998). Consistent with this result, *AUX1* and *AXR1* were upregulated at concentrations of Cd and As(III) that promoted root hair elongation (**Figure 3**). In addition, their expression was decreased in *gem* plants, which have shorter root hairs (**Figure 8**), and not affected in *ttg1 Arabidopsis*, which has a higher root hair number (**Figure 6**). These results confirmed that *GEM* is also involved in root hair elongation. In accordance with our results regarding *AUX1*, a previous report indicated that Cd stress induces *AUX1* expression in rice root hairs, and the root hair length in *osaux1* mutant rice is shorter than that of the WT (Yu et al., 2015). The involvement of auxin in root hair elongation is supported by our results showing increased levels of auxin in the root tip in parallel with the increased root hair length caused by Cd and As(III) (**Figure 4C**).

Ethylene signaling components also seem to play a role in root hair elongation, since the ethylene insensitive mutant *ein2* has shorter hairs (Rahman et al., 2002), while the ethylene overproducing mutant *eto1* has longer root hairs (Strader et al., 2010). In support of this, the ethylene signaling components *ETR1* and *EIN2* were upregulated in response to Cd and As(III), stimulating root hair elongation (**Figure 3**). Consistent with this result, the negative regulator of ethylene signaling *CTR1* was downregulated by Cd and As(III). In the case of *ttg1* plants showing an increase in root hair density, the expression of ethylene signaling genes was not altered (**Figure 6**). By contrast, ethylene signaling genes were repressed in *gem* plants, which display a reduction in root hair density and length (**Figure 8**). Regarding the relationship between ethylene and auxin, ethylene is able to enhance auxin synthesis and transport (Ruzicka et al., 2007; Swarup et al., 2007), and ethylene promotes root hair elongation via auxin-induced microtubule randomization (Takahashi et al., 2003). In addition, ethylene may be involved in root hair initiation, since ACC (ethylene precursor) enhances root hair number (Tanimoto et al., 1995) and AVG (ethylene biosynthesis inhibitor) reduces root hair number (Masucci and Schiefelbein, 1996).

It is also possible that Cd promotes root hair elongation by increasing ROS production, which activates root hair cell elongation (Takeda et al., 2008). This hypothesis is supported by reports that root hair length was induced by Cu (Pasternak et al., 2005) and Cr (Castro et al., 2007), which are associated with changes in ROS metabolism and auxin homeostasis.

It is also suggested that Cd enhances root hair formation by inducing Fe deficiency through the inhibition of Fe uptake, since Fe deficiency promotes root hair production and elongation by enhancing ethylene production (Schmidt and Schikora, 2001). Cd reduces Fe uptake by competing for the iron transporter *IRT1* (Vert et al., 2002).

During the last decades, dynamic gene regulatory network (GRN) models have been used extensively to investigate cell fate determination (von Dassow et al., 2000; Albert and Othmer, 2003; Espinosa-soto et al., 2004; Almaas, 2007). These models integrate experimental data and produce a dynamic model to explore developmental patterns. Such models are potentially useful and can improve our knowledge regarding biological developmental systems, since they provide powerful tools to interpret contrasting data. In addition, new hypotheses can arise from these models that can be experimentally validated. For instance, Benitez et al. (2007) used a dynamic model to show that positional *SCM* signals in the cortex can function either positively or negatively on *WER* and *CPC* expression within the active or inactive complex, respectively. Although *WER* expression is proposed to be repressed by the SCM signal (Kwak et al., 2005), the possibility of a direct effect on *CPC* could not be excluded (Wada et al., 1997; Lee and Schiefelbein, 2002; Costa and Dolan, 2003; Benitez et al., 2007). In the model presented here, we attempted to show how Cd and As (III) affect the GRN in root epidermal cells based on the transcriptional levels of the genes examined. Our model (**Figure 9**) is only a schematic integration of the active molecules in each cell type. Moreover, this does not include the complete GRN model components within each cell type, in contrast to dynamic models presented in recent studies (Benitez et al., 2008; Kwak and Schiefelbein, 2008; Savage et al., 2008). Information derived from dynamic GRN studies (Benitez et al., 2007, 2008; Kwak and Schiefelbein, 2008; Benitez and Alvarez-Buylla, 2010) can improve our understanding of cell fate determination and patterning, as well as possible interactions within the GRN and cell to cell communication in *Arabidopsis* root epidermal cells. In this context, the GRN model can not only predict and provide novel insights into GRN component interactions involved in root hair patterning in response to Cd and As, but also help link heavy metal signaling pathways and genetic regulatory network components under different experimental condition in future studies.

For further experiments, the conversion of non-hair cell to hair cells (change of fate) induced by Cd and As(III) should be examined using morphological analyses. In addition, the expression of various fate-determining genes in response to metals, such as *WER*, *GL3*, *EGL3*, *CPC1*, *TRY*, and *ETC1*, should be examined. Analysis of the effects of metals on recently identified genes expressed in hairy and non-hairy cells is also meaningful (Bruex et al., 2012).

## Conclusion

The density and length of root hairs are increased in response to Cd and As(III) in *Arabidopsis* by the following mechanisms: First, root hair fate determination (differentiation) is stimulated through the upregulation of GEM (positive regulator) and the downregulation of TTG1/GL2 (negative regulators). Second, the initiation of root hair is promoted by the upregulation of RHD6. Third, the elongation of root hair is promoted by increasing auxin content and the expression of the auxin signaling genes *AXR2*, *AUX1*, and *AXR1*. Finally, root hair elongation is stimulated by the upregulation of the ethylene signaling genes *ETR1* and *EIN2*, and the downregulation of *CTR1*.

## Author Contributions

SH designed and supervised whole research, and wrote the article with contributions of all the authors; RB and DK equally performed most of the experiments; JK performed an initial experiment.

## Conflict of Interest Statement

The authors declare that the research was conducted in the absence of any commercial or financial relationships that could be construed as a potential conflict of interest.

## Footnotes

1 http://rsb.info.nih.gov/ij

## Abbreviations

ACC: 1-aminocyclopropane-1-carboxylic acid
ANOVA: analysis of variance
AsNaO2: Arsenate sodium
AVG: aminoethoxyvinylglycine
CdSO4: Cadmium sulphate
EDTA: Ethylene diamine tetra acetic acid
GUS: β-glucuronidase
MS: Murashige and Skoog
qRT-PCR: quantitative RT-PCR
ROS: Reactive oxygen species
WT: Wild type
X-gluc: 5-bromo-4-chloro-3-indolyl-β-D-glucuronidase
