# Supplementary material for: The Density and Length of Root Hairs Are Enhanced in Response to Cadmium and Arsenic by Modulating Gene Expressions Involved in Fate Determination and Morphogenesis of Root Hairs in Arabidopsis
Source: Front Plant Sci. 2016 Nov 23;7:1763. doi: 10.3389/fpls.2016.01763 (PMC5120091; doi:10.3389/fpls.2016.01763)
Supplement: Supplementary file 1 [file Data_Sheet_1.pdf]

Supplementary Material

The density and length of root hairs are enhanced in response to cadmium and arsenic by modulating gene expressions involved in fate determination and morphogenesis of root hairs in *Arabidopsis*

Ramin Bahmani, DongGwan Kim, JinA Kim and Seongbin Hwang*

*** Correspondence:** Corresponding Author: sbhwang@sejong.ac.kr

1 Supplementary Figures and Tables

Supplemental Figure S1. Effect of Cd and As (III) on expressions of genes (semi-quantitative RT-PCR) involved in the differentiation (fate determination) and elongation of root hairs in *Arabidopsis*.

Supplemental Figure S2. Effect of Cd and As (III) on expressions of genes (semi-quantitative RT-PCR) involved in the differentiation (fate determination) and elongation of root hairs in *ttg1 Arabidopsis*.

Supplemental Figure S3. Effect of Cd and As (III) on expressions of genes (semi-quantitative RT-PCR) involved in the differentiation (fate determination) and elongation of root hairs in *gem Arabidopsis*.

Supplemental Figure S4. Effect of Cd and As (III) on root epidermal cell length in *Arabidopsis*.

Supplemental Table S1. Sequence of primers used for gene expression analysis using Real-Time PCR.

Supplemental Table S2. Sequence of primers used for gene expression analysis using Semi-quantitative RT-PCR

1.1 Supplementary Figures

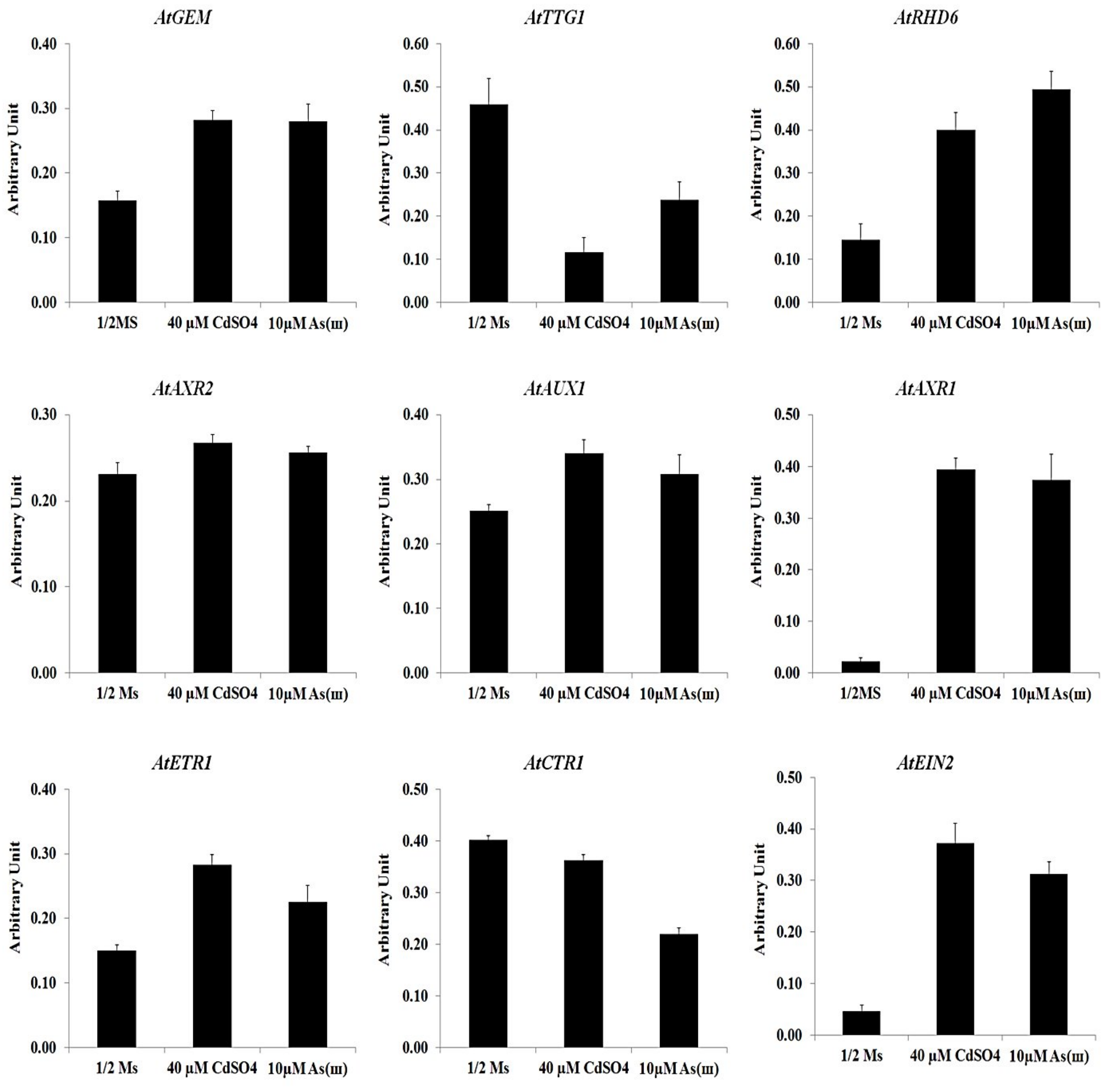

Supplemental Figure S1. Effect of Cd and As (III) on expressions of genes (semi-quantitative RT-PCR) involved in the differentiation (fate determination) and elongation of root hairs in *Arabidopsis*. *GL2* graph is not included since *GL2* expression was not detectable on the gel.

1.2 Supplementary Figures

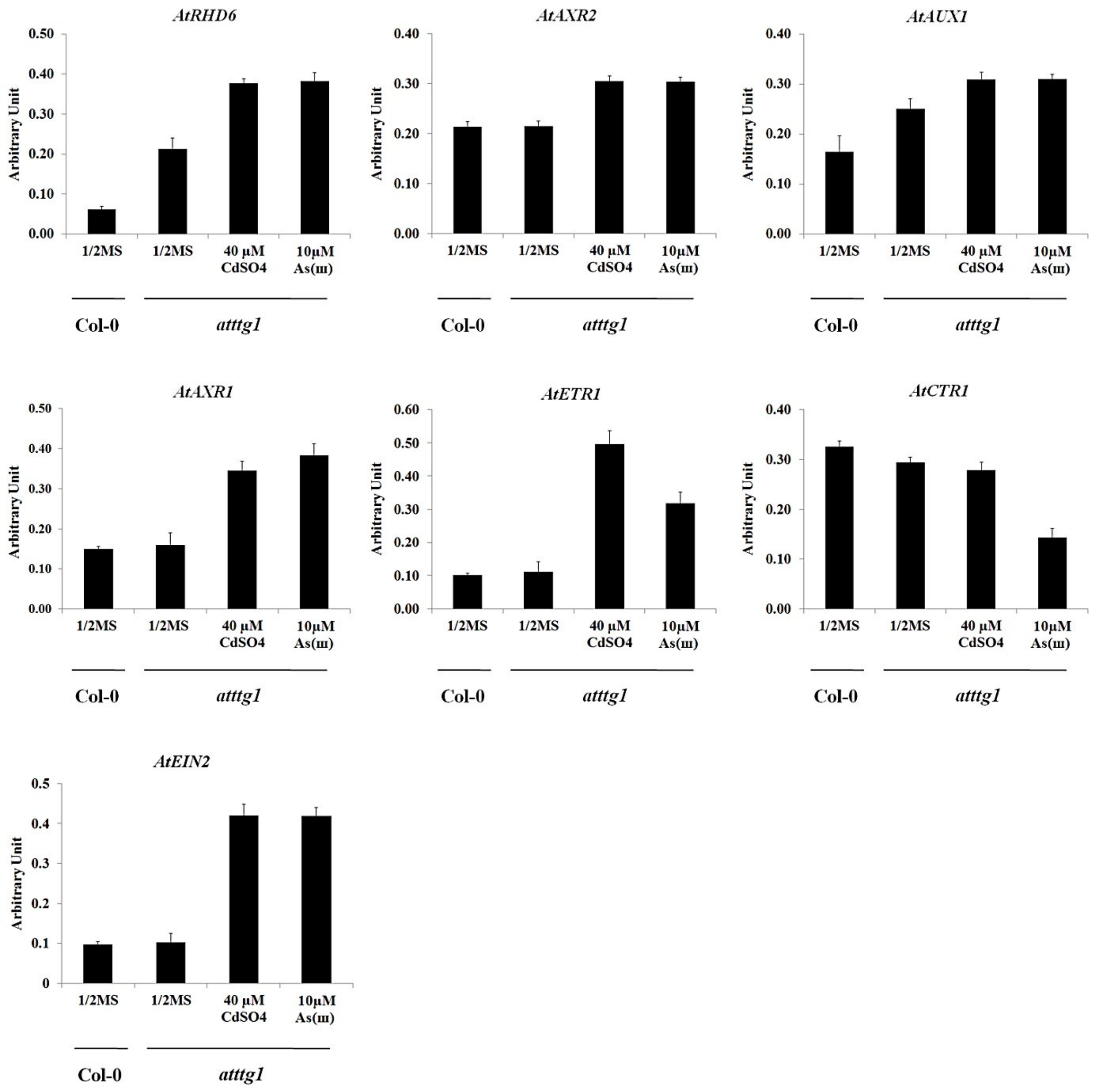

Supplemental Figure S2. Effect of Cd and As (III) on expressions of genes (semi-quantitative RT-PCR) involved in the differentiation (fate determination) and elongation of root hairs in *ttg1 Arabidopsis*. *GL2* graph is not included since *GL2* expression was not detectable on the gel.

1.3 Supplementary Figures

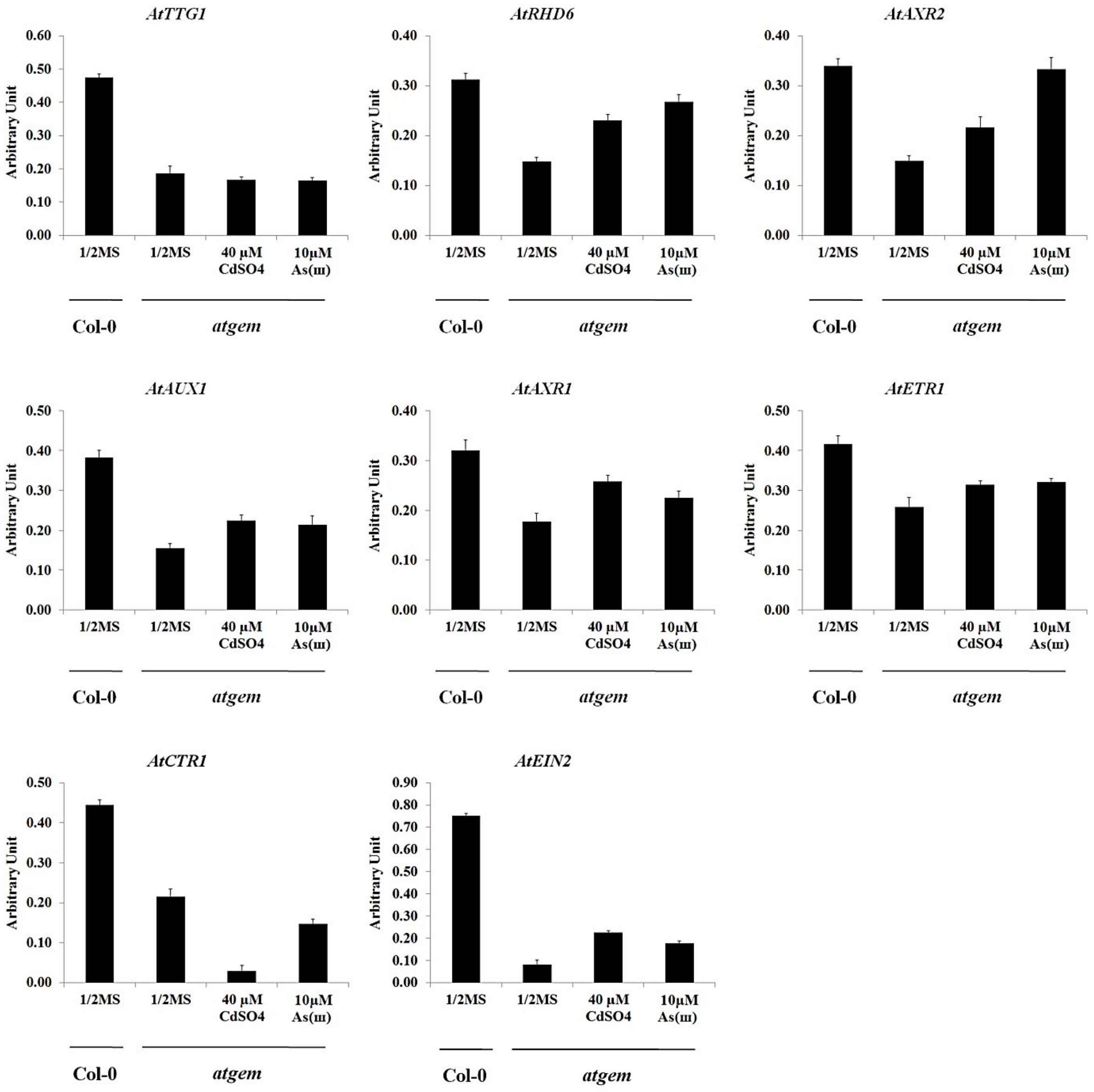

Supplemental Figure S3. Effect of Cd and As (III) on expressions of genes (semi-quantitative RT-PCR) involved in the differentiation (fate determination) and elongation of root hairs in *gem Arabidopsis*. *GL2* graph is not included since *GL2* expression was not detectable on the gel.

1.4 Supplementary Figures

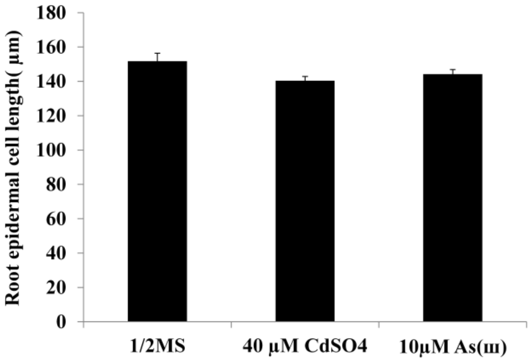

Supplemental Figure S4. Effect of Cd and As (III) on root epidermal cell length in *Arabidopsis*

1.5 Supplementary Tables

Supplemental Table S1. Sequence of Real-Time PCR primers used for gene expression analysis in this study.

Gene name	Locus	Primer Sequences
AtActin	AT3G18780	F: 5′-GCAGAGCGGGAAATTGTAAG-3′<br>R: 5′-TTCTCGATGGAAGAGCTGGT-3′
AtGEM	AT2G22475	F: 5′-TGCTTGGTAGATGGGGAAAG-3′<br>R: 5′-ATTCTTCCCATTGCAGCATC-3′
AtTTG1	AT5G24520	F: 5′-CGAGCATCCTTATCCTCCAA-3′<br>R: 5′-GCTCGACGGTTGATGAATCT-3′
AtGL2	AT1G79840	F: 5′-ACGTGGGACAATGGAAAGAG-3′<br>R: 5′-AGCTGCATCTCTCCGAACAT-3′
AtRHD6	AT1G66470	F: 5′-CCTAAATCCGCTGGAAACAA-3′<br>R: 5′-TTGTTGGCTTAGGCTTGGTC-3′
AtAXR2	AT3G23050	F: 5′-GAACTTTGGTGGAGGAGCAG-3′<br>R: 5′-GGCCAATGCATCAGAAAGAT-3′
AtAUX1	AT2G38120	F: 5′-CCACTCCAACGCTTTCTCTC-3′<br>R: 5′-TGTCATGCATCCCAATCACT-3′
AtAXR1	AT1G05180	F: 5′-AACGGCTCAGTACTCCCAGA-3′<br>R: 5′-CGGAACAAACTGCTTTGTGA-3′
AtETR1	AT1G66340	F: 5′-GTACACCTTGGGTGCGAAGT-3′<br>R: 5′-CGTGAATACGGAGAGCGATT-3′
AtCTR1	AT5G03730	F: 5′-CGGTCACTCAACCTCCAAAT-3′<br>R: 5′-ACTCAGGCGACGTCTCTCAT-3′
AtEIN2	AT5G03280	F: 5′-ACAAGGAGGAACGAAGACGA-3′<br>R: 5′-TCGAACGGATTTCTTGATCC-3′

1.6 Supplementary Tables

Supplemental Table S2. Sequence of primers used for gene expression analysis using Semi-Quantitative PCR.

Gene name	Locus	Primer Sequences
AtActin	AT3G18780	F: 5′- GATATTCAACCAATCGTGTG -3′<br>R: 5′- GGATACTTCAAGGTAAGAATAC -3′
AtGEM	AT2G22475	F: 5′-ATG GAG CCG CCG AAG GGA GAT-3′<br>R: 5′- CAC ACC GAC CTT AAA GCA CCG -3′
AtTTG1	AT5G24520	F: 5′- ATG GAT AAT TCA GCT CCA GAT TC -3′<br>R: 5′- CAA ACT CTA AGG AGC TGC ATT T -3′
AtGL2	AT1G79840	F: 5′-ACGTGGGACAATGGAAAGAG-3′<br>R: 5′-AGCTGCATCTCTCCGAACAT-3′
AtRHD6	AT1G66470	F: 5′- ATG GCA CTC GTT AAT GAC C -3′<br>R: 5′- TAA TTG GTG ATC AGA TTC GA -3′
AtAXR2	AT3G23050	F: 5′- ATG ATC GGC CAA CTT ATG AAC -3′<br>R: 5′- CAA GAT CTG TTC TTG CAG TAC -3′
AtAUX1	AT2G38120	F: 5′- ATG TCG GAA GGA GTA GAA GC -3′<br>R: 5′- TCA AAG ACG GTG GTG TAA AG -3′
AtAXR1	AT1G05180	F: 5′- CGA TGC GAA GAG TGT TGG -3′<br>R: 5′- GTA AGT CCC CAA CAT CGG -3′
AtETR1	AT1G66340	F: 5′- ATG GAA GTC TGC AAT TGT ATT G -3′<br>R: 5′- TTA CAT GCC CTC GTA CAG TAC -3′
AtCTR1	AT5G03730	F: 5′- ATG GAA ATG CCC GGT AGA AG -3′<br>R: 5′- ACA AAT CCG AGC GGT TGG C -3′
AtEIN2	AT5G03280	F: 5′- CAC TTG GCT CAG ATC TGC -3′<br>R: 5′- CAT ATG CAC CCA ACA TCT G -3′
